# Preparation and Application of Decellularized ECM-Based Biological Scaffolds for Articular Cartilage Repair: A Review

**DOI:** 10.3389/fbioe.2022.908082

**Published:** 2022-06-30

**Authors:** Qian Zhang, Yixin Hu, Xuan Long, Lingling Hu, Yu Wu, Ji Wu, Xiaobing Shi, Runqi Xie, Yu Bi, Fangyuan Yu, Pinxue Li, Yu Yang

**Affiliations:** ^1^ Department of Orthopedics, The Second People’s Hospital of Guiyang, Guiyang, China; ^2^ Department of Obstetrics and Gynecology, Affiliated Hospital of Guizhou Medical University, Guiyang, China; ^3^ Senior Department of Orthopedics, Forth Medical Center of Chinese PLA General Hospital, Beijing, China; ^4^ School of Medicine, Nankai University, Tianjin, China

**Keywords:** extracellular matrix, mesenchymal stem cells, tissue engineering, decellularization, articular cartilage regeneration

## Abstract

Cartilage regeneration is dependent on cellular-extracellular matrix (ECM) interactions. Natural ECM plays a role in mechanical and chemical cell signaling and promotes stem cell recruitment, differentiation and tissue regeneration in the absence of biological additives, including growth factors and peptides. To date, traditional tissue engineering methods by using natural and synthetic materials have not been able to replicate the physiological structure (biochemical composition and biomechanical properties) of natural cartilage. Techniques facilitating the repair and/or regeneration of articular cartilage pose a significant challenge for orthopedic surgeons. Whereas, little progress has been made in this field. In recent years, with advances in medicine, biochemistry and materials science, to meet the regenerative requirements of the heterogeneous and layered structure of native articular cartilage (AC) tissue, a series of tissue engineering scaffolds based on ECM materials have been developed. These scaffolds mimic the versatility of the native ECM in function, composition and dynamic properties and some of which are designed to improve cartilage regeneration. This review systematically investigates the following: the characteristics of cartilage ECM, repair mechanisms, decellularization method, source of ECM, and various ECM-based cartilage repair methods. In addition, the future development of ECM-based biomaterials is hypothesized.

## 1 Introduction

**FIGURE 1:**
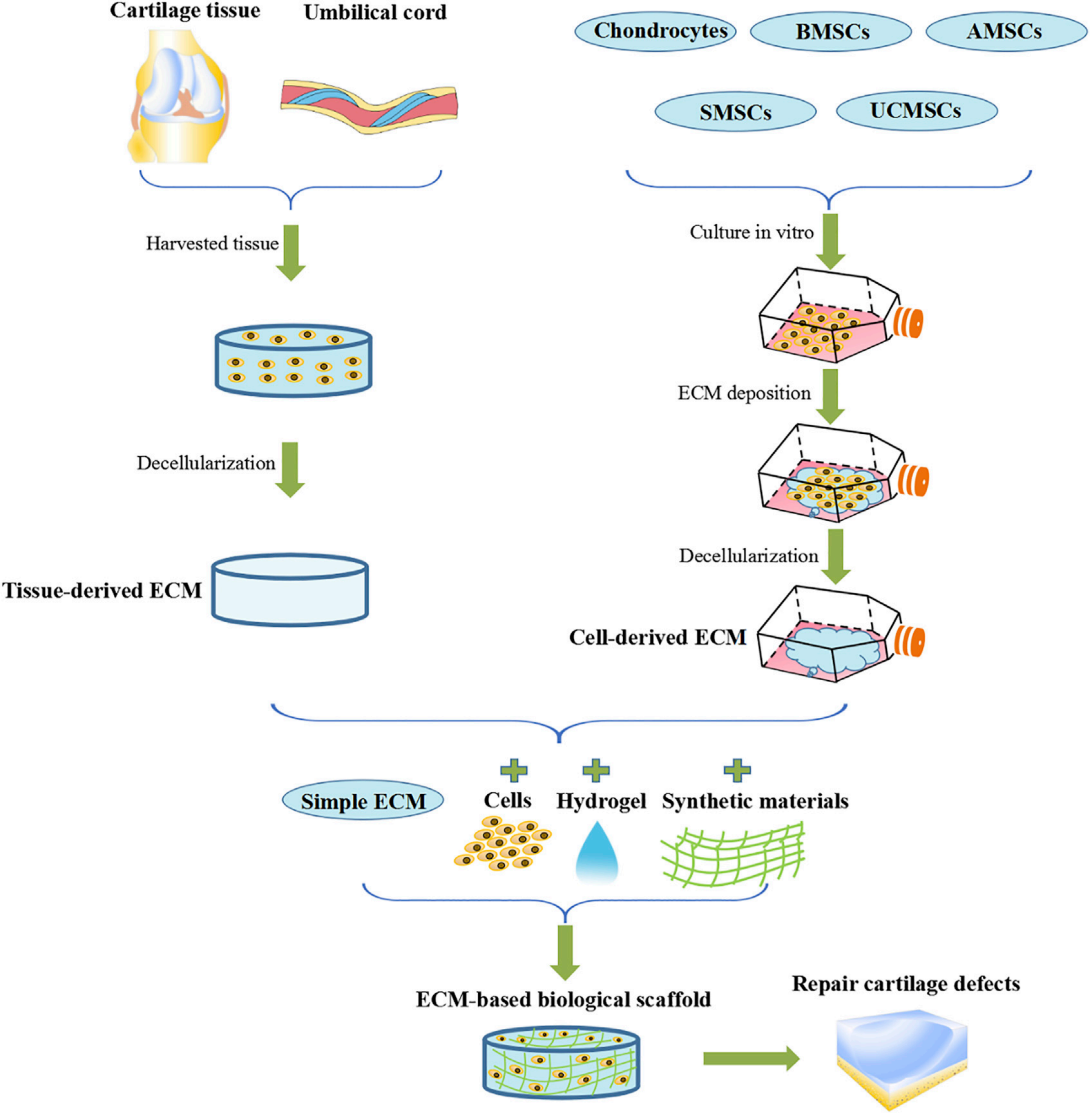
ECM-based biological scaffolds for AC defect repair.

Articular cartilage (AC) is a hydrated viscoelastic connective tissue that does not have innervation, lymphatic contraction or blood flow. It is composed of low levels of chondrocytes (∼1–5% of the total tissue volume), which are surrounded by compact anti-adhesion extracellular matrix (ECM). These chondrocytes are extremely poor at proliferating at a rate of almost zero. As a result, AC rarely regenerates or repairs itself after damage or degeneration caused by common diseases such as osteoarthritis (Camarero-Espinosa et al., 2016). Currently, the repair of AC injury includes conservative treatment and surgical treatment. Conservative treatment is mainly to relieve pain and inflammation through drugs, including nonsteroidal anti-inflammatory drugs (NSAIDs), cyclooxygenase 2-selective (COX-2) inhibitors and articular cavity injection of corticosteroids (Yang et al., 2020). And there are three common AC regeneration techniques used in clinics including microfracture (MF) (Kraeutler et al., 2020), autologous chondrocyte implantation (ACI) (Gille et al., 2016) and autologous/allogeneic cartilage transplantation (Hangody et al., 1997; Frank et al., 2018). Although widely used and effective, these methods all possess various limitations and disadvantages (Richter et al., 2016) (Table 1). Contrastingly, tissue-engineered AC shows superior benefits (Fu et al., 2020). Tissue engineering technology is mainly to obtain seed cells through *in vitro* isolation and culture, and then inoculate them into scaffolds to construct tissue engineering repair materials and implant AC defects for repair (Krafts, 2010; Eftekhari et al., 2020). Currently, scaffolds used in tissue engineering are biomimetic prepared from natural materials and synthetic materials according to the structure and composition characteristics of AC ECM (Li et al., 2021). The use of ECM-based AC biological scaffolds as cartilage repair scaffold materials provides mechanically supportive macroscopic and microscopic environment. This promotes regeneration of the structure and function of the AC (Swinehart and Badylak, 2016). However, the highly unique, complex structure of AC ECM, as well as its multiple components mean that it cannot be simulated by any material. As a result of these unique characteristics, cartilage ECM has superior advantages as a potential scaffold material for the repair of AC defects. This review discusses the potential use of ECM-based biomaterials for AC defect repair (Figure 1). In doing so, various topics are systematically reviewed, this includes characteristics of AC ECM, repair mechanisms, decellularization method, source of ECM, and various ECM-based AC repair methods.

**TABLE 1:** Comparison of cartilage defect treatments widely used in clinics.

Treatment	Defect Area	Disadvantages
MF	<2 cm^2^	Regenerated cartilage is mainly composed of fibrous cartilage
ACI	>2 cm^2^	Two operations required, limitations in donor site, frequent donor site complications, uneven cellular distribution in receptor site, frequent cellular loss, unstable cellular phenotypes
Autologous cartilage transplantation	<4 cm^2^	Limitations in donor site, donor site complications, graft is not matched to the defect
Allogeneic cartilage transplantation	>4 cm^2^	Requirement to sustain chondrocyte viability, high standard donor age and challenges in preserving grafts

## 2 Structural Composition and Function of AC ECM

Articular cartilage is a unique connective tissue composed of chondrocytes surrounded by dense ECM. Moving from superficial to deep, AC ECM is divided into four areas. These are the superficial, middle, deep, and calcified layer (Vega et al., 2017). Each of these four areas possess great differences in chondrocyte morphology, cell phenotype, and ECM composition (Johnstone et al., 2013). AC ECM is secreted and regulated by chondrocytes. It’s main components are collagen and proteoglycan, which are mixed with various bioactive factors, including growth factors, integrins, and functional peptides. Collagen and proteoglycan are expressed differently in different cartilage regions. Collagen concentrations decrease gradually moving from the superficial to the deep layer of AC. The opposite pattern is reported for proteoglycans (Carballo et al., 2017). This multi-layered regional structure is formed as a result of the hydrodynamic factors continuously applied to the cartilage interface. This occurs from bone development to maturity and creates the unique tough and elastic mechanical properties of cartilage (Camarero-Espinosa and Cooper-White, 2017). Collagen, mainly type II collagen, is largely responsible for the maintenance of cartilage structure and elastic strength. However, the role of proteoglycan in the fibrin reticular structure is to maintain articular cartilage viscoelasticity and flexibility (López-Ruiz et al., 2016).

To date, the mechanisms via which ECM scaffolds promote AC regeneration have not yet been fully elucidated. However, it is known that ECM-derived scaffolds do in fact exert some positive effects on AC regeneration. The potential mechanisms and roles of ECM in promoting AC regeneration are discussed in this section.

### 2.1 Cell-ECM Interactions

Chondrocytes are present in AC tissue in low levels and are scattered throughout. As a result, direct communication or interaction between chondrocytes is infrequent. Interactions between the cells and ECM play an important role in maintaining AC ECM homeostasis. This also forms the main signaling pathway controlling AC formation (Peng et al., 2021).

The ECM controls cell outcome by binding to cell surface receptors. Cell surface receptors, including transmembrane integrins, recognize specific ECM fragments. Integrins play a key regulatory role in the development of cell-specific tissues. Integrins not only promote physical interactions between cells and ECM, but also initiate intracellular signal transduction, and promote cytoskeletal reorganization. What’s more, they can alter cell survival, growth, movement and differentiation, as well control responses to mechanical stimulation (Muncie and Weaver, 2018).

Cell products, including proteases, exert a modifying effect on the ECM. Contrastingly, the growth factors and cytokines bound to the ECM play a more functional role. These growth factors and cytokines control the metabolic and secretory activity of these cells. However, looking specifically at the AC microenvironment, interactions between the cells and ECM remain a dynamic process. Biomechanical stimuli can influence this process. This reciprocal dynamic process reciprocity is essential in maintaining the normal function of AC. Bidirectional signal transduction between cells and the ECM can control cell function. Cells and ECM can fuse to each other, and subsequently maintain tissue homeostasis. This occurs via a subtle balance of two-way intracellular mechanical conduction signals (from outside to inside) and ECM mechanics (from inside to outside) (Hynes, 2009). On one hand, cells replenish the ECM and reshape it to adapt to changes in the surrounding environment. On the other hand, the modification of ECM can also impact the phenotype and behavior of cells (Miller et al., 2020).

### 2.2 Regulating Proliferation and Chondrogenic Differentiation

The ECM can anchor and regulate the fate of mesenchymal stem cells (MSCs) through its own specific physical (specifically matrix stiffness and mechanical force) and biochemical (specifically its matrix binding growth factor) properties (Lane et al., 2014; Chua et al., 2016). A well-characterized ECM can be designed as a highly bioactive and functional scaffold for tissue regeneration, cancer therapy and other fields (Fu, 2021).

The mechanical properties and interface morphology of ECM are key signal factors of cell proliferation, differentiation, migration and apoptosis (Prein and Beier, 2019). Effectors such as matrix stiffness and mechanical forces imposed by these factors influence cell regulation and tissue regeneration (Avenoso et al., 2018). The normal cell cycle regulates the replication of stem cells and somatic cells, and the cells proliferate rapidly during physiological processes such as tissue repair and embryogenesis. Cell-cell and cell-matrix interactions largely determine the degree of cell proliferation. Apoptosis occurs when adherent cells fail to adhere to the cell surface (Sella et al., 2018). Cell physical interactions with ECM regulate cell cycle and cell death through integrin-dependent cell signaling, which regulates G1 and M phases of the cell cycle. Integrins bind to a large number of ECM proteins, most notably type II and VI collagen and fibronectin that initiate signals in response to mechanical forces (Prein and Beier, 2019). Chondrocyte integrins can also be used as mechanical sensors (Prein and Beier, 2019). In addition, cell proliferation is enhanced when cells are subjected to mechanical tension from ECM. Cell surface receptors transfer mechanical tension to intracellular actin cytoskeleton. It has been shown that ECM-induced increased focal adhesion kinase (FAK) activity leads to active Rho/Rho kinase signal transduction, leading to high actin cytoskeleton tension, which further promotes cell proliferation (Schrader et al., 2011). In addition to enhancing proliferation, physical stimulation also could regulate the ability of MSCs to differentiate. Studies have shown that simulated soft gelatinous brain tissue, elastic muscle tissue and bone surface matrix induce neurogenic, myogenic and osteogenic phenotypes respectively (Engler et al., 2006). Shear forces from ECM induce differentiation of MSCs while altering their structure (Jaasma and O'Brien, 2008). The contraction of the actin cytoskeleton mediated by the Rho signaling pathway is important for the osteogenic differentiation of MSCs and further enhances RUNX2 expression (Patwari and Lee, 2008). It is well known that osteogenic differentiation is reviewed prior to chondrogenic and adipogenic induction (Rodríguez, 2004). With the maturation of the surrounding environment and the adaptation of the cytoskeleton, appropriate physiological signals such as shear force and hydrostatic pressure will gradually enhance the chondrogenesis of MSCs (Steward et al., 2013).

Biomaterials based on AC ECM are capable of stimulating the natural AC environment via the provision of adhesion sites and biochemical cell signaling. These actions can help recruit and differentiate MSCs to promote AC regeneration. There are various reports in literature describing the intracellular signal transduction pathway of the interaction between ECM and MSCs (Watt and Huck, 2013). Various biochemical components of the ECM control the proliferation and differentiation of MSCs via the interaction of ECM and integrins (Sart et al., 2020). Various bioactive factors in AC ECM possibly play an important role in the differentiation process of MSCs into chondrocytes. Studies have reported that GAG contained in AC ECM is beneficial to cell signal transduction as well as cell infiltration migration. Type II collagen, chondroitin sulfate, proteoglycan, and other proteins promote the stem cell differentiation into chondrocyte. This can be achieved through integrin-mediated signal transduction (Sutherland et al., 2015; Tamaddon et al., 2017). What’s more, hyaluronic acid interacts with cell surface receptors (specifically CD44 and CD168) to induce both stem cell migration and AC differentiation. Soluble transforming growth factor-β (TGF-β) in AC ECM promotes MSCs differentiate into cartilage. In addition, insulin-like growth factor-1 (IGF-1) and TGF-β3 promote the induction of TGF-β via the transcriptional regulation of cartilage-specific genes (Benmassaoud et al., 2020).

### 2.3 Store for Various Bioactive Factors

ECM is the deposit for various specific tissue bioactive factors, including growth factors. The ECM regulates their spatial location, stability as well as their biological activity (Huang et al., 2017). ECM macromolecules and soluble signal molecules chelate via non-covalent interactions. As an example, TGF-β1 and bone morphogenetic protein-2 (BMP-2) bind in this manner to type II collagen (Zhu and Clark, 2014).

Various soluble factors are found in the ECM, including TGF-β (Murdoch et al., 2007; Madry et al., 2014; Coricor and Serra, 2016; Ying et al., 2018), BMP (Murphy et al., 2015; Deng et al., 2018; Gomez-Puerto et al., 2019), IGF-1 (Frisch et al., 2014; Zhou et al., 2016; Liebesny et al., 2019), FGF (fibroblast growth factor) (Pizzute et al., 2016a), and GDF-5 (growth and differentiation factor-5) (Coleman et al., 2013). These factors can influence the proliferation of cells and differentiation of cartilage. They play an integral role in cell proliferation, cell differentiation, and maintenance of cell-specific phenotypes. Interestingly, after decellularization, ECM retains some of its bioactive factors, which subsequently influence the exogenous and endogenous cells belonging to the ECM scaffold.

## 3 Preparation of the ECM Scaffold

### 3.1 AC Tissue Decellularization

**FIGURE 2:**
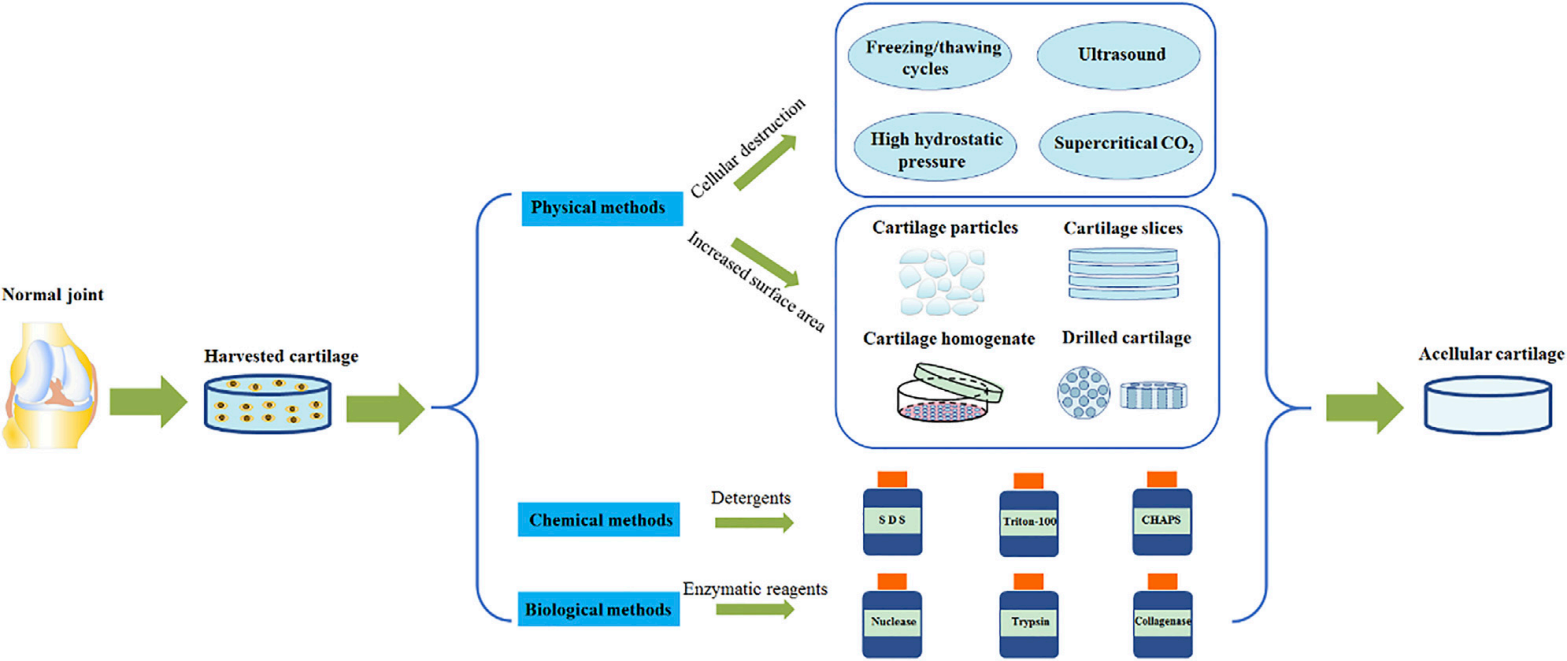
AC tissue decellularization.

AC tissue decellularization uses detergents to diffuse into the cartilage tissue space and cleave chondrocytes. Cell fragments and genetic materials can be subsequently washed away. As a result of the density of cartilage, it is often challenging for detergents to fully permeate. A combination of physical, chemical and biological methods is mainly used to aid this process (Figure 2). Moreover, the characteristics of different AC decellularization methods are summarized (Table 2). The ultimate goal is to achieve a fine balance between the removal of all immunogenic residues and retaining biochemical components. Crapo et al., proposed a minimum standard for the degree of decellularization. This standard dictates three points, firstly that the content of dsDNA in ECM per mg dry weight is less than 50 ng, secondly, that the length of the DNA fragment is less than 200 bp and finally that there is no nuclear component detectable by either HE or DAPI tissue section staining (Crapo et al., 2011).

**TABLE 2:** Characteristics of different AC decellularization methods.

Methods	Principles	Advantages	Disadvantages	References
Physical	Using physical principles to cleave the cells and destroy any cell matrix adhesion proteins	1. Convenient	1. Inadequate decellularization efficiency	Pulver et al. (2014), Hiemer et al. (2016), Hiemer et al. (2019), Chen et al. (2021), Shen et al. (2020)
		2. Low immune response	2. Destroying the ultrastructure of ECM	
		3. Low toxicity		
		4. Maintaining part of the structure		
Chemical	Using chemical detergents to destroy the structure of cell membrane and separate DNA from proteins, removing cellular substances from the tissue	1. High decellularization efficiency	1. Reducing specific component content and bioactivity of ECM	Gawlitta et al. (2015), Schneider et al. (2016), Gilpin and Yang, (2017), Browe et al. (2019), Ghassemi et al. (2019), Luo et al. (2019)
		2. Retaining the structure and composition of ECM to a large extent	2. High toxicity of residual chemicals	
Biological	Using enzymatic reagents to remove cell residues and tissue components	1. Removal of residual cells and antigens specifically	1. Longer processing time	Rana et al. (2017), Kim et al. (2018)
		2. Less damage to other bioactive components	2. Immune response caused by residual enzyme reagents	

#### 3.1.1 Physical Methods

Physical methods to destroy cells have one main purpose: to use physical principles to cleave the cells and destroy any cell matrix adhesion proteins. The most commonly used methods are summarized in the following paragraphs.

The most frequently used method is harnessing the powers of the freeze/thaw cycle. The freeze/thaw cycle destroys cells via the formation of ice crystals, which stimulate cell rupture (Pulver et al., 2014). Through this method, protein loss in the ECM is minimal and the mechanical properties are not affected. However, the ultrastructure of the ECM is destroyed, and as a result further removal of cell debris is needed (Pulver et al., 2014; Roth et al., 2017). Ultrasound waves can be used to release chondrocytes from the cavity (Shen et al., 2020). This method does not use a decontamination agent and enables retention of the GAG composition and cartilage structure. However, this method only suitable for cartilage slices less than 30 μm (Shen et al., 2020). AC can also be treated at high hydrostatic pressure (HHP) to inactivate cells (Hiemer et al., 2016; Hiemer et al., 2019). This occurs as water molecules enter the protein complex, and maintaining the structure and biomechanical properties of AC ECM. What’s more, supercritical CO_2_ extraction technologies can be employed to remove cell debris in AC tissue (Chen et al., 2021), maintain scaffold structure, and retain the type II collagen composition. All with the added benefit of using no chemical reagents.

In addition, some physical methods are used to prepare ECM by increasing the penetration of chemical agents (Zhang et al., 2020). These methods mainly treat AC tissues by physical mechanical treatment, such as pulverizing AC tissues into particles (Liebesny et al., 2019) or homogenizing after slicing (Almeida et al., 2017; Luo et al., 2019). This enables the chemical reagent to better access the dense central matrix of cartilage, and as a result achieves an enhanced cellularization effect. Other methods include drilling of AC via mechanical force (Zhang et al., 2019a) and carbon dioxide laser technology (Li et al., 2019). Both of these methods increase the surface area of the AC tissue, which facilitates improved detergent penetration of detergent, improved degree of acellularity, and enhanced cell adhesion. It is important to note that although these physical processing techniques can facilitate detergent penetration into the dense AC tissue, they also destroy the unique heterogeneous structure of AC tissue (Ghassemi et al., 2019).

#### 3.1.2 Chemical Methods

Various types of detergents (for example ionic, non-ionic and zwitterionic detergents) are effective acellular chemical reagents. These detergents destroy the structure of cell membrane, separating DNA from proteins and remove cellular substances from the tissue (Das et al., 2019; Das et al., 2020). The concentration and speed of action of the reagent will have an important impact on the following: its acellular effect, the structure and composition of the AC ECM, macrostructure destruction, decreases in the GAG content, and the change of micromechanical properties.

##### 3.1.2.1 Ionic Detergents

Sodium dodecyl sulfate (SDS) dissolves cells and their membranes (Schneider et al., 2016), and is more efficacious at nuclear removal than other types of detergent. SDS also significantly reduces proteoglycan levels and damages the mechanical properties of tissue (Browe et al., 2019; Ghassemi et al., 2019).

##### 3.1.2.2 Non-Ionic Detergents

Triton-100 is a mild decontamination agent, which removes residual DNA in tissue via the destruction of lipid-lipid and lipid-protein structures (Xia et al., 2019). Triton-100 is often used in combination with ammonium hydroxide. In comparison to SDS, the damage caused by Triton-100 to AC structure is less severe. In fact, the content of AC regulatory protein I and growth factor was higher after acellular decellularization, whereas glycosaminoglycan levels are reduced (Luo et al., 2019).

##### 3.1.2.3 Zwitterionic Detergent

CHAPS (3-[(3-cholamidopropyl) dimethylammonium]-1-propanesulfonate) is a zwitterionic surfactant and performs excellently in maintaining tissue protein and structure. CHAPS is however inefficient at removing DNA from residual cells (Gawlitta et al., 2015; Gilpin and Yang, 2017).

There is evidence that non-ionic and zwitterionic ionic detergents have better effect on maintaining matrix ultrastructure in various chemical methods (Hudson et al., 2004). Nevertheless, chemical methods still have some drawbacks. For example, an immune response caused by residual chemical detergents could destroy collagen fibers and other protein components in ECM (Meezan et al., 1975; Meyer et al., 2006), so it is necessary to fully remove any residues. In addition, the mixed use of a variety of chemical detergents has greater damage to proteins in ECM (Alhamdani et al., 2010).

#### 3.1.3 Biological Methods

Various enzymatic reagents (including nuclease, trypsin, and collagenase) are commonly used to remove specific undesirable cell residues and tissue components. However, these residual enzymes can potentially damage regenerating cell viability and induce immune responses. In addition, ethylenediamine tetra-acetic acid (EDTA) is often used as a chelating reagent. EDTA can chelate divalent cations, destroy the attachment of cells to collagen via Arg-Gly-Asp receptors, and facilitate the dissociation of cells from ECM proteins. This improves the efficiency of acellular decellularization (Rana et al., 2017). A combination of an enzymic reagent and a chelating agent, such as trypsin and EDTA, is frequently used to selectively destroy cell adhesion proteins on the carbon side of arginine and lysine (Kim et al., 2018).

### 3.2 Sources of ECM

ECM is a complex mixture of structures and molecules with various biological properties. Molecules are arranged in a highly unique three-dimensional topological pattern.

ECM-based biological scaffolds are taken via acellular treatment of specific tissues. Currently, the acellular methods employed are mainly physical, chemical and biological. These methods are used to remove the maximum amount of cellular material, whilst preserving the complex components and three-dimensional structure (Aamodt and Grainger, 2016). Acellular decellularization methods differ depending on the tissue characteristics. There are various factors determining the chosen method. These include tissue thickness, lipid content, tissue cellularity, and cell density. Each acellular method will alter the ECM composition and destroy the structure of ECM. However, the realistic aim is to minimize these adverse effects rather than avoid them completely (Crapo et al., 2011). An ideal approach would be to combine the advantages and disadvantages of the various decellularization methods and create an optimal tissue disruption method. This method would create an ECM free of cells and genetic material, whilst retaining its specific structure, composition, and biomechanical characteristics essential to its function.

#### 3.2.1 Decellularized Native Tissue

##### 3.2.1.1 ECM Derived From AC Tissue

ECM derived from AC tissue maintains its natural structure and inherent components. This can induce chondrogenic cells to develop towards chondrogenic direction via the promotion of cell proliferation and differentiation (Luo et al., 2015; Rowland et al., 2016; Rothrauff et al., 2017a; Rothrauff et al., 2017b).

Yang et al. used physical crushing, chemical acellular, freeze-drying and cross-linking to transform human joint AC into ECM scaffolds derived. The resultant scaffold has an interconnected structure, which promotes the migration of cells to pores. This structure also facilitates the transport of nutrients and metabolic waste, and enhances the communication between cells in different pores. What’s more the authors induced the differentiation of canine bone marrow MSCs (BMSCs) into chondrocytes *in vitro*. They also co-cultured these cells with the ECM scaffold and implanted the co-culture in nude mice subcutaneously. This protocol successfully produced ectopic AC-like tissue (Yang et al., 2008). Yin et al. crushed fresh goat knee joint AC using physical force. They then chemically decellularized the mixture. After screening for specific sizes of AC particles, the resultant rat BMSCs were combined with the AC particles. Using the rotating cell culture system Culture, *in vitro* experiments confirmed that the acellular AC particles promoted the adhesion and proliferation of BMSCs and induced the differentiation of BMSCs into AC without the addition of exogenous growth factors. *In vivo* experiments, micro-tissue aggregates formed on acellular AC particles promoted the regeneration of rat femoral trochlear cartilage. As a result, high-quality hyaline AC tissue was produced (Yin et al., 2016). Subsequently, the authors used similar methods to supplement rabbit articular chondrocytes and adipose MSCs (AMSCs) into porcine AC ECM constructs. This method confirmed that the mixture promoted adhesion and proliferation of chondrocytes and AMSCs. This method also verified the enhanced phenotype of chondrocytes as well as the fact that AMSCs were induced to differentiate into AC. Importantly, this protocol successfully repaired AC defects in the rabbits examined (Yin et al., 2018).

##### 3.2.1.2 ECM Derived From Umbilical Cord Wharton Jelly

The purpose of elastic Wharton jelly is to protect umbilical cord blood vessels from external pressure. UCWJ has relatively few cells and lacks the structures of blood vessels, nerves and lymphatics. Interestingly, UCWJ is rich in collagen, glycosaminoglycans, hyaluronic acid, and various growth factors. These features make UCWJ very similar to natural AC ECM (Baba et al., 2013; Corrao et al., 2013). Zhao et al. used both physical and chemical methods to decellularize human UCWJ. The aim of this was to prepare porous oriented AC scaffold materials. These materials promote the adhesion, orientation and proliferation of the chondrocytes implanted on the scaffold. This methodology was applied to *in vivo* experimentation and resulted in the successful repair of AC defects in rabbit knee joints (Zhao et al., 2018). Safari et al. also confirmed that acellularized ECM scaffolds derived from the human umbilical cord promote chondrogenic differentiation by providing a natural environment for human BMSCs (Safari et al., 2019).

##### 3.2.1.3 A Comparison of ECM Derived From AC Tissue and UCWJ

In a recent study, Xiao et al. prepared human UCWJ-ECM and porcine knee AC ECM scaffolds (Xiao et al., 2017). They subsequently implanted rabbit chondrocytes on these two respective scaffolds. After *in vitro* culture, the two scaffolds were analyzed and cross comparisons were performed. Both scaffolds were identified to be hydrophilic, have porous orientation structure. No significant differences in pore size and porosity were identified. However, both scaffolds showed a reasonable affinity for chondrocytes and were able to simulate the natural ECM microenvironment and therefore were able to promote the adhesion and proliferation of chondrocytes. Importantly, in comparison to AC tissue ECM scaffolds, UCWJ-ECM scaffolds showed stronger biomechanical properties, contained more growth factors (such as IGF-I, TGF-β), and showed high levels of type II collagen and GAG gene expression. The AC tissue ECM scaffold was shown to be stronger than the UCWJ-ECM scaffold at promoting the chondrocytes proliferation (Xiao et al., 2017). This comparative study illustrates the superiority of UCWJ-ECM scaffolds over AC tissue ECM scaffolds. The structure of natural AC is dense and acellular reagents struggle to fully permeate. This means it is difficult to decellularize. What’s more, AC tissue ECM scaffolds cannot provide enough internal space for cell penetration and proliferation (Jin et al., 2007). UCWJ is the main component of umbilical cord, and its sources are abundant. Preparing ECM scaffolds is simple to perform, and there are minimal ethical challenges. Taking together the evidence from these studies, there is potential to replace ECM scaffolds of AC tissue in the near future. Consequently, these scaffolds could be more widely used as AC tissue engineering materials.

Tissue-derived ECM is usually obtained via decellularization from allogeneic or xenogeneic tissues or organs. This acellularized tissue can recruit MSCs or progenitor cells from the bone marrow or synovium of the joint. This allows these cells to migrate through the gaps between tissue fragments as well as within cavities left in the tissue after removal. Biological materials of these origins have been approved by the FDA and are widely used as tissue engineering materials. However, it is important to note that these materials have some shortcomings that pose difficulties for widespread application. This includes the transmission of pathogens, inflammation and anti-host immune response. What’s more, the degradation rate is variable, and difficult to control (Liao et al., 2010; Skóra et al., 2012).

#### 3.2.2 Decellularized Cultured Tissue

Decellularized cultured tissue refers to ECM secreted by deposited chondrocytes/MSCs and then obtained by decellularization techniques (Pizzute et al., 2016b; Liu et al., 2016). Cell-derived ECM possesses more advantages than tissue-derived ECM. These include a lower probability of pathogen transmission, lower levels of inflammation or anti-host immune responses, and most importantly, a higher similarity with the microenvironment of natural ECM. Its structure is relatively loose and has desirable porosity. In addition, decellularized cultured tissue is not limited by insufficient of cell penetration or proliferation of tissue-derived ECM during the recellularization process (Lu et al., 2011). Interestingly, various types of chondrogenic cells, including chondrocytes and MSCs, are used as decellularized cultured tissue to support cell proliferation and AC differentiation (Sun et al., 2018).

##### 3.2.2.1 Chondrocytes Derived ECM

Wang et al. cultured rabbit chondrocytes *in vitro* to form cell sheets. They also obtained chondrocyte-derived ECM via chemical decellularization. *In vitro* experimentation showed that the chondrocytes in the ECM sheets were cleanly removed and therefore maintained the natural structure of ECM. As a result, they possessed enhanced ability to migrate BMSCs. The expression of SOX-9 was increased and the expression of COL-X was decreased. The AC sheets created successfully repaired the AC defects of rabbit knee joint, without adding exogenous cells (Wang et al., 2018).

The ECM secreted by chondrocytes cultured *in vitro* is comparable to that of natural AC tissue. Both have the same microenvironment and the ability to recruit host endogenous cells. This can promote stem cell proliferation, cartilage formation, and differentiation. There is no requirement for exogenous cell implantation, which has a beneficial effect on AC regeneration (Jin et al., 2007; Jin et al., 2010). It is important to note that during the *in vitro* culture of chondrocytes, it is challenging to prevent hypertrophy and dedifferentiation of chondrocytes. This means the normal phenotype of chondrocytes is frequently lost. In addition, in the majority of cases, only young donors have primary chondrocytes capable of producing high quality AC ECM (Hoshiba et al., 2012). This makes it challenging to obtain sufficient numbers of donors (Tottey et al., 2011). In addition, *in vitro* chondrocyte culture it is difficult to avoid hypertrophy and differentiation of chondrocytes. This results in the loss of the normal chondrocytes phenotype (Chen and Kawazoe, 2018).

##### 3.2.2.2 MSCs Derived ECM

MSCs derived from connective tissue (including bone marrow, fat, and the umbilical cord) have outstanding differentiation potential in adipogenic, osteogenic, and chondrogenic lineages. What’s more, they have been widely used in tissue engineering. ECM derived from MSCs can be obtained relatively easily from cultured MSCs via acellular treatment. The ECM contains a variety of paracrine and autocrine factors (for example TGF-β and BMP-2). These factors play an active role in maintaining chondrocyte phenotypes (Barker, 2011; Zhu, 2020). MSC-derived ECM are capable of fully expressing the niche of stem cells, protecting the cells inoculated on the ECM scaffold from oxidative stress damage, as well as further promote cell proliferation (Assunção et al., 2020; Xing et al., 2020).

In another study, Wang et al. obtained ECM scaffolds from rabbit BMSCs after SDS acellular treatment. The study reported that ECM scaffolds provide a superior microenvironment for MSCs which is less immunogenic by nature. The scaffold was shown to facilitate the regeneration of osteochondral defects in rabbit knee joints (Wang et al., 2020). Yang et al. also reported that ECM derived from human BMSCs acted as a substrate in chondrocyte proliferation and phenotype maintenance, and promoted chondrocyte redifferentiation. The study also verified that ECM derived from human BMSCs could be used as a carrier for chondrocyte implantation (Yang et al., 2018). Tulin et al. inoculated human AMSCs into the acellular ECM scaffold derived from human AMSCs (via physical and chemical methods). The cells were successfully integrated into the porous ECM scaffold with high levels of cell viability and proliferation ability. AC specific proteins, namely type II collagen and aggrecan, were synthesized after culture *in vitro*. With an extended culture time, the content of GAG increased. This confirmed differentiation of AMSCs into AC (Ibsirlioglu et al., 2020). Yan et al. used Triton-100 combined with ammonium hydroxide to decellularize cell slices derived from rabbit knee joint synovial mesenchymal stem cells (SMSCs). They then used the ECM as an *in vitro* expansion system for rabbit articular chondrocytes. This ECM was shown to improve the proliferation abilities of the chondrocytes whilst also enhancing their anti-inflammatory properties. This study reported this ECM to be an excellent culture substrate for *in vitro* chondrocyte expansion (Yan et al., 2020). Zhang et al. obtained ECM derived from human umbilical cord-deposited mesenchymal stem cells using a combined method of chemical and biological digestion. The three-dimensional culture of rabbit articular chondrocytes with this ECM confirmed that the material is an appropriate tissue-specific niche for chondrocytes. This ECM significantly promoted the proliferation of chondrocytes and enhanced their differentiation ability (Zhang et al., 2019b).

## 4 Repair Strategies Using ECM Biological Scaffolds

### 4.1 ECM Based-Scaffolds Alone

Tang et al. generated rabbit and pig autologous BMSCs-derived ECM scaffolds. They combined these two sources of scaffolds with bone marrow stimulation technology with the aim of repairing rabbit femoral trochlea and pig medial femoral condyle AC defects. These respective animal models confirmed that ECM scaffolds derived from autologous BMSCs increased the number of BMSCs in bone marrow stimulating exudate. This stimulated AC repair and recovery (Tang et al., 2019).

### 4.2 Combination of Cells and ECM-Based Scaffolds

Jia et al. added bovine knee AC into ECM scaffolds with a longitudinal oriented structure and the elastic modulus of the scaffold was showed 3 times higher compared to non-oriented scaffold. Next, the authors induced rabbit BMSCs into chondrocytes *in vitro* and these cells were inoculated onto the scaffold. After sustained culture, the cells proliferated significantly. They arranged along the pores of the oriented scaffolds and adhered uniformly to the scaffold pore walls. This cell-scaffold composite successfully repaired rabbit knee AC defects. What’s more, this longitudinally oriented ECM scaffold resulted in significantly enhanced biological properties of regenerated AC *in vivo* (Jia et al., 2012; Jia et al., 2015). Porcine AC-derived ECM scaffold was prepared by Zhang et al. using Human UCWJ-MSCs and goat primary chondrocytes implanted on the scaffold. In this experiment, the primary chondrocytes retained the original chondrocyte phenotype. The UCWJ-MSCs maintained a stable state of AC differentiation. Excitingly, the scaffold complex successfully repaired AC defects in goat knee joints (Zhang et al., 2020).

### 4.3 Combination of Hydrogels and ECM-Based Scaffolds

Hydrogels have been used in AC tissue engineering. This is largely because of their characteristics including easy molding, adjustable mechanical properties, excellent cytocompatibility, biodegradability, and appropriate biological activity. ECM is dissolved and processed to form the hydrogels. Some natural ECM structures and signal transduction substances are preserved in the hydrogels. This influences the phenotype, proliferation, migration and differentiation of cells (Saldin et al., 2017).

Bordbar et al. produced hydrogels from sheep knee AC after both physical and chemical treatment. The rabbit BMSCs contained within the hydrogel were adhesive, capable of proliferation, and capable of differentiation into chondrocytes (Bordbar et al., 2020). After culturing human BMSCs in bovine AC-derived hydrogels *in vitro*, Rothrauff et al. detected AC-specific gene expression (namely Sox9, Aggrecan, and Type II collagen) (Rothrauff et al., 2018). Beck et al. supplemented rat BMSCs to porcine AC derived hydrogels. This resulted in upregulated expression of chondrogenic genes and promotion of matrix synthesis (Beck et al., 2016).

### 4.4 Combination of Synthetic Materials and ECM-Based Scaffolds

AC tissue engineering materials are required to create a suitable natural microenvironment for AC regeneration. In addition to this, they must also have the biomechanical properties enabling them to resist the stress of joint movement. Notably, ECM scaffolds possess poor mechanical properties. Contrastingly, synthetic polymers exhibit superior mechanical properties. They do however lack inherent biological activity. Synthesizing a combination of the two materials could create a superior AC repair material.

In their study, Zheng et al. exploited porcine knee AC ECM to simulate the natural AC biochemical components. The authors used poly (lactide-co-glycolide) (PLGA) to enhance the mechanical strength of ECM scaffolds. They also prepared composite scaffolds with a specifically oriented structure. The biomimetic composite scaffold was shown to have acceptable hydrophilicity, porosity and orientation. It was also shown to have preferable adhesion and proliferation effect on rabbit BMSCs. Most importantly, its mechanical strength was shown to be been greatly improved (Zheng et al., 2011). On the basis of this study, Guo et al. implanted autologous BMSCs on a composite scaffold, with the aim of repairing rabbit knee AC defects. The regenerated AC tissue mirrored the structure of natural AC. The repair effect was desirable (Guo, 2018). Stocco et al. designed a novel scaffold combining UCWJ-ECM and polyvinyl alcohol (PVA) hydrogel. The scaffold promoted the adhesion of chondrocytes whilst maintaining the phenotype of chondrocytes (Stocco et al., 2014). Xu et al. added ECM derived from rabbit chondrocytes on the surface of PCL electrospinning to form composite scaffolds. This nanoscale scaffold was reported to be suitable for the expansion of rabbit BMSCs and have acceptable chondrogenic ability (Xu et al., 2021).

## 5 Discussion

ECM has a complex 3D network structure and is composed of hydrated macromolecular proteins and sugars. Various soluble factors are bound to ECM, interactions with these factors form the acellular matrix microenvironment of the tissue. The ECM-based tissue repair process is comparable to normal tissue development and growth. As a part of normal tissue repair, ECM degrades and synthesizes continuously. ECM reaches a state of equilibrium, which promotes a cycle of tissue repair. This equilibrium results in a change in the structure and composition of ECM. This structural change is particularly evident in terms of collagen and proteoglycan levels (Cai et al., 2017). The ECM has a complex dynamic macro- and micro-environment with desirable biomechanical, biochemical and biophysical properties. It not only mimics the natural framework and attachment site, but also contains natural and intrinsic biological elements. This includes adhesion ligands, topological characteristics, and mechanical resistance. These elements play a role in cell-ECM bidirectional signal transduction, cell homeostasis, proliferation, migration, differentiation, and regulation of cell gene expression (Zhang et al., 2016). Researchers have proposed using ECM in addition to the three elements of tissue engineering (these are widely accepted as scaffolds, cells, and growth information). This information verifies that ECM plays a pivotal role in tissue repair and regeneration.

As an avascular tissue, cartilage tissue is considered to have an “immune privilege” that does not easily trigger host immunity (Li et al., 2022). Few researchers have investigated the immunogenicity of ECM materials derived from allogeneic or xenogeneic cartilage. In fact, the immune response is necessary for tissue regeneration to some extent, because biomaterials can modulate the immune response and create a more favorable microenvironment for tissue reconstruction (Brown and Badylak, 2013). The immune response induced by ECM materials depends on the degree of decellularization in part. Due to the inadequate decellularization efficiency of some physical methods, the immune response of ECM obtained by them is mainly cellular. Residual cellular components in ECM may cause problems related to cellular compatibility, and some may even lead to chronic inflammation (Brown et al., 2009). On the other hand, chemical and biological methods can trigger a specific immune response to residual reagents. In addition, ECM material also has certain immunomodulatory ability. It has been widely demonstrated that AC ECM can directly regulate the macrophage phenotype to exert immune effects and achieve better cartilage regeneration (Wei et al., 2021; Li et al., 2022). Usually, Continuous polarization of M1-type (pro-inflammatory) macrophages can impair tissue repair, while biomaterials with high pro-M2-type (anti-inflammatory and pro-repair) macrophage polarization ability can achieve ideal regeneration (Sicari et al., 2014). Tian et al. found that porcine articular cartilage derived ECM promoted the transformation of macrophage phenotype from M1-like macrophage population to M2-like macrophages in a rat knee osteochondral defect model (Li et al., 2022). In general, ECM has a low immunogenicity and can play a role in regulating immune function to offset the negative effects of allotransplantation to a certain extent. With advances in decellularization methods and subsequent treatments, ECM with more appropriate immunogenicity is expected.

AC exists in a complex environment surrounded by many types of cells. These cells include AMSCs, BMSCs, synovial MSCs and et al. (Mendelson et al., 2011). Due to their biological characteristics, ECM-based scaffold materials are able to recruit endogenous stem cells to the site of AC injury (Agrawal et al., 2010). Under the action of various environmental factors (including growth factors, joint fluid, and mechanical stimulation of joint activity), stem cells can be induced into chondrocytes. This subsequently promotes AC tissue regeneration.

The design and research of various natural and synthetic materials to simulate the natural structure and composition of ECM for use in AC engineering is a hot topic (Benders et al., 2013). Although materials (including collagen and chitosan) are natural components of AC ECM and possess some biological advantages, they are inferior compared to the specific environment of AC ECM. Synthetic materials (including polylactic acid and polyglycolic acid) can simulate the structure and composition of AC ECM to the greatest extent possible via artificial intervention. However, their biggest downfall is their poor biocompatibility.

Natural derived ECM has a favorable host response and integration ability, but it is rapidly absorbed in the process of AC repair. This factor renders the effects as short-term and inhibits any long-term benefit as a scaffold. Synthetic polymers are artificially made into an ordered structure consisting of mesh and fibers. The polymers are then combined with natural ECM, which joins the chemical stability of the synthetic polymer and the biological compatibility of natural ECM (Shu et al., 2013). This combined method of “strength and strength” undoubtedly creates a promising new path and paves the way for the development of tissue engineering AC materials. The properties of this composite can be adjusted via manual intervention. Properties such as porosity, mechanical strength, and degradation rate, can be controlled. 3D printing can even be used to create a specific layered structure (Xing et al., 2020). According to requirements, seed cells, growth factors, cytokines and other factors can also be added to the composite material. This can ensure sufficient signal transduction and biological recognition ability. This can also ensure the regeneration of completely natural high-quality hyaline AC tissue.

## 6 Conclusion and Prospective

Currently, tissue engineering technology has made unprecedented leaps forward, and a variety of tissue engineered AC repair materials have been derived. Learning from the unique properties of a wide range of materials, special composite materials have been produced. ECM materials derived from suitable sources are fused with composites to creatively obtain new materials that are closely consistent with the cartilage/osteochondral layered structure and unique chemical composition of the defect. This process can promote extracellular matrix deposition, cell adhesion, growth factor release, and receptor signal transduction. This can enable regeneration of tissue consistent with natural cartilage/osteochondral. These techniques have been widely used in a large number of basic, pre-clinical, and clinical trials.

With the rapid development of medicine, material science, and biological printing technology, material technology no longer meets the needs of tissue engineering repair. Time is a crucial factor in all stages of cell proliferation, migration, and differentiation. Developing an enhanced understanding of how the cellular microenvironment evolves over time is key. Multidimensional simulations of the cellular microenvironment can solve this mystery. We predict that on the near horizon, ECM-based biological scaffolds which adapt to their environment over time, will become a hot topic in AC repair tissue engineering research.
